# The Hygienic and Dietetic Management of Phthisis

**Published:** 1894-11

**Authors:** L. P. Barbour

**Affiliations:** Thomasville, Ga., Formerly Professor of Diseases of Children and Lecturer on Physical Diagnosis, Medical Department of the University of the South; Member of the Tennessee State Medical Association; of the Tri-State Medical Society of Tennessee, Georgia and Alabama, &c., &c.


THE
### Southern Medical Record.

A MONTHLY JOURNAL OF MEDICINE AND SURGERY.

VOL. XXIV. ATLANTA, GA., NOVEMBER, 1894. No. 11.

### Original Articles.

### THE HYGIENIC AND DIETETIC MANAGEMENT OF PHTHISIS.

BY L. P. BARBOUR, M. D., THOMASVILLE, GA.,

Formerly Professor of Diseases of Children and Lecturer on Physical Diagnosis, Medical Department of the University of the South; Member of the Tennessee State Medical Association; of the Tri-State Medical Society of Tennessee, Georgia and Alabama, &c., &c.

We hope we are in the dawn of a brighter day as regards the successful treatment of phthisis. The discovery of the specific germ is bearing fruit, and we may yet have a specific remedy. But such a remedy, be it ever so destructive of baccilli, can never supercede careful hygienic management. No disease is more insidious in its advance, more variable in its conditions, more protean in points of attack. A thorough study of every individual case, with watchfulness in every detail of hygienic management, always will be, as it always has been, of utmost importance.

An experience of several years, both as physician and as a patient, at health resorts frequented by consumptives, has shown me that there is lamentable carelessness on the part of many in the profession in this respect. Patients are sent from home in a vain search for health, at boarding houses with poor food, impure water and bad surroundings; or at fashionable hotels where there is overmuch gaiety and dissipation. Other patients, though having good food and good surroundings, offset these advantages by imprudence in diet, by staying closely in badly-ventilated rooms, or by over-exercise. Still others fail to gain as they should by insufficient exercise.

Now this is all wrong. And it is in the hope that a presentation of the proper hygienic management of phthisis, briefly told, may help to overcome the carelessness and indifference of so many, and result in better care of some of these sufferers, that this paper is written.

First we will give the right management of an average case not too far advanced for hope—then we will discuss special symptoms.

### CLIMATE AND OUTDOOR LIFE.

That climate is best where the patient's general health is best, and that is usually where the patient may live an outdoor life, provided it is not too warm. A very cold country taxes the vitality of a patient too much if he is kept out of doors. A very warm climate enervates. An occasional frosty morning with bright, cheery days, such as South Georgia offers in winter, is as near the ideal as we can attain. A very dry climate might be good, except for the intolerable dusts of such places. This dust is directly irritant to the diseased lungs, and must be avoided. So here, again, the medium condition is to be preferred.

Every patient, wherever he is sent, should be disabused of the idea that there is a specific climate. The fact that climatic treatment is only a part of the general hygienic management should be thoroughly impressed upon his mind, so that he may not offset a good climate by otherwise bad hygiene. There should be no "trusting simply to climate."

The particular resort should be selected with a view to good drainage, pure water supply, and good hotels. Here the patient should live out of doors as much as possible. Living out of doors does not mean that he must be hunting or fishing, or climbing mountains all the time. Exercise adapted to his condition having been taken, the rest of the time is to be spent lounging in easy chairs on sunny verandahs. If the weather is very cool, let him be sufficiently protected by overcoat and wraps and still kept out of doors. Even patients that are quite weak and unable to take any exercise can be thus wrapped up and kept out most of the time. The extremities should especially be kept warm. Rooms occupied by patients should be well-lighted and well-ventilated, the air being kept as pure and fresh as that out of doors. This is possible in properly constructed houses. Many people from the North keep their rooms over-heated. This is to be avoided. On the other hand, many Southern houses cannot be sufficiently warmed during a cold snap. All these things should be investigated in selecting a boarding place.

In country towns the water-closets are usually situated in the back yards, often at some distance from the house. Aside from the usual unhygienic condition of such places, the exposure necessary to reach them during stormy weather is a decided detriment to phthisical patients. Modern hotels and boarding houses, with the water-closet under the same roof, are greatly to be preferred.

WATER.

I have already referred to the importance of a pure water supply. Especially should care be taken that the patient is not using water infected with disease germs. An attack of dysentery or of typhoid fever may turn the scale from victory to defeat. Typhoid fever—the so-called mountain fever—is not rare at many resorts east and west. Those places are fortunate which have a water supply from artesian wells.

Little or nothing is gained in the ordinary case, by the use of mineral waters. That water is best which is freest from all impurities, organic and inorganic.

DIET.

It is not wise to keep a patient on a closely limited diet. Tiring of the few things allowed, he does not take sufficient nourishment. A convenient plan is to have printed slips containing a list, first of the things which should form the chief part of his diet; second, of the things which may be taken carefully, and in small quantity; and, third, of the things to be avoided.

The first list will consist largely of easily digested proteïd substances. Tender broiled steak, roast beef, roast lamb, mutton chops, properly cooked eggs, sweet milk, butter milk, custards, etc., etc., together with light bread, toast, well-cooked rice, oat-meal, cracked wheat, soups, etc. In the second list we may put fruits, a larger variety of vegetables, fish, etc. The more indigestible foods, as salt meats, salt fish, cabbages, beans, etc., are to be avoided. These lists must of course be modified to suit individual cases. One patient, in an early stage, with considerable vigor, good appetite and good digestion, may be given much liberty; while a feeble one with weak digestion must be carefully watched. Such as the latter may need very light meals, both as to quantity and digestibility, and milk given between meals and at bed-time. If the milk is badly borne, it may be given predigested, or bouillon, or clam broth used. A cup of clam broth given during the night often aids in bringing refreshing sleep.

Most patients need instructions as to how to drink milk; it is not to be gulped down, but taken in sips, fifteen minutes being spent over each glass. Time and again I have had people tell me they could not drink milk, only to find it agreed well when properly taken.

Sometimes a glass of light wine, or of beer, taken with meals, enables a patient to eat more, and is in this way beneficial. But if used at all, they should be prescribed as medicines, and exact directions given as to how much is to be taken and how often. The indefinite advice to "take whiskey," is a source of great harm. Stomachs are ruined by too much, and no good whatever accomplished.

### REST AND EXERCISE.

While myself a patient, and after I had sufficiently recovered to take considerable exercise with advantage, it was a matter of astonishment to me to meet so many debilitated patients walking over the hills and riding horse-back. Engaging in conversation with these. I often inquired what professional instruction they had received. A usual answer was: "My doctor told me to come here and take all the outdoor exercise possible."

It was a piteous sight—these patients adding to the burden of a weakened heart, already overtaxed by an infiltrated lung, by climbing hills or clinging to a horse till utterly exhausted.

The advice to be outdoors is proper enough, but patients with fever should be told to take as *little* exercise as possible, rather than as much as possible. And even those with little or no temperature, and with considerable vigor, should be carefully taught to never exert themselves until fatigued, never to take exercise severe enough to cause violent heart action, and never to get out of breath.

But *instructions* as to how to take care of themselves do not suffice. Few patients have judgment and self-control sufficient to manage their own case properly. The usual hopefulness of a consumptive leads him to exaggerate a slight improvement, and to overestimate his own strength. Patients should always be under intelligent supervision, the effect of exercise upon the temperature and upon the heart noted, and the amount and kind of exercise regulated accordingly. A very few require urging to take sufficient exercise; the majority need restraining.

A form of exercise which gives pleasure is of course more valuable than exercise for its own sake; for this reason hunting and fishing would be commended. But the excitement of such sport is sure to lead the patient to overdo, and not infrequently, to harmful exposure. Hence, except for the few in a very early stage, these things must be condemned. Bicycle riding, rowing and tennis playing are also to be prohibited because of the severe exertion.

Judgment is required in deciding what patients may take a little exercise, and what ones should take none, and when, in the process of recovery, others may begin. Some with an afternoon fever, can take a carriage ride in the morning with advantage. As the temperature lessens and strength increases, they can begin short walks, lengthening the distance with returning health. Many people require instructions as to how to walk for healthful pleasure and profit. Not at a break-neck speed, as if intent on having it soon over, but slowly, saunteringly, observing everything about them and stopping frequently to listen to the song of a bird, or to gather a handful of wild flowers.

Mountain climbing must be left for those with a good deal of vigor, and then done slowly and with frequent rests.

All forms of exercise must be followed by periods of rest. Usually these can be spent in the open air; but for some the quietude of their room is best. Most patients are benefited by an hour or two of rest in their room, with sleep if possible, during the afternoon. By this means the mind is rested with the body, and mental rest is as important as physical rest. So also is mental over-exertion and worry as harmful as physical over-exertion. All sources of excitement must be avoided; all study, and especially all business cares must be avoided if the patient is to have the best chance possible.

Sleep, nature's restorer, is to be sought by all possible hygienic means, and drugs left as a last resort. Regular hours and quietude are two important aids to this end, and should be remembered in selecting a boarding place. Pure air in the bedroom is an essential to perfect rest.

BATHING.

Nothing is gained by special mineral or electric or thermal baths. But in the cold sponge-bath we have a most excellent tonic. It should be given by an attendant and followed by vigorous rubbing. This is the most effectual treatment we have to protect the system against changes of temperature and susceptibility to colds. The baths should be given in the morning just before the patient arises; an arm being bathed and rubbed till thoroughly dried and all aglow, then the chest, the other arm, etc., the rest of the body being well wrapped in woolen blankets meanwhile. After the bath is over, the patient should stay in bed until thoroughly warm and comfortable. In this way ice water can generally be used, but if it leaves a sense of chilliness and depression, the cold water must be abandoned and lukewarm water used instead. This is gradually made cooler each morning until very cold water is resumed. Of course those in an advanced stage, with little vitality, cannot be expected to use cold water. Sponging in warm or hot water with brisk rubbing afterwards, adds to the comfort of such.

A few words now as to special symptoms.

### COUGH.

When we have instituted such hygienic, dietetic and medicinal measures as are best adapted to overcome the tubercular process, we have done the best thing for the cough, and all, as a rule, that should be done. Especially should a cough with free expectoration be let alone. Occasionally a cough is so persistent and harassing as to keep the patient from securing sufficient sleep. If due to throat complication, it should receive appropriate local treatment; if to tenacious bronchial secretion, inhalations may relieve; finally, we may be compelled to prescribe some simple opiate, preferably codeine, that the patient may rest.

### HEMORRHAGE.

*Rest* is the chief indication in the hygienic management of pulmonary hemorrhage. If it is a comparatively slight hemorrhage of an early stage, rest is all that is needed. If it is a severer hemorrhage due to the invasion of large vessels by the destructive process, it is best treated by the application of ice over the heart and over the seat of hemorrhage, combined, of course, with perfect rest and appropriate medicinal treatment. Hemorrhages are often the result of over-exercise, and are, therefore, much less frequent in those under careful control.

### FEVER.

To overcome the long continued fevers of phthisis, whether that fever is due to the tubercular process, or to septic infection, we must conserve the vital forces most carefully and reinforce in all ways possible. When we permit a patient whose tissues are rapidly being consumed by temperature, whose lung surface is not sufficient for aeration of the blood except during comparative quietude, whose heart is already laboring excitedly to drive the impoverished blood through pulmonary obstruction—when we permit such a patient to take active exercise, we are certainly not conserving but increasing waste and destruction. I have seen just such cases out walking and riding.

Even though there be very little destruction of lung tissue, still must rest be enforced when the temperature is high, if we would guard the heart, prevent further destruction, and repair damages already done. Whoever heard of a typhoid patient being told to "walk off the fever?" Yet it is just as reasonable as for the patient with phthisis to attempt it. Rest is clearly the first hygienic indication in this as in all other fevers. On pleasant days, part of the time may be spent on cots in the open air, but as near absolute rest as possible must be enforced if the temperature goes very high. Those cases which have considerable strength and a temperature at or near the normal during the first half of the day, may without harm spend their mornings indulging in social games and light reading; but, as soon as the temperature reaches a hundred, they should take to cots or to their beds. Some of these may be allowed to take short drives during the morning remission, or even short walks if their condition is otherwise favorable and the temperature never rises much above one hundred. But when exercise of any kind is permitted a fever patient, he must be watched carefully for its effects upon heart and temperature.

The diet of fever patients should be easily digested and nutritious, the meals light and frequently repeated. Some cases need to be confined to liquid foods—predigested milk, peptonized meats, beef juice, malted milk, buttermilk, broths, etc. Others with low morning temperatures and better digestion, can take quite substantial breakfasts of beef-steak, oat meal, light-bread, etc., but need the liquid diet the rest of the day while the temperature is high.

Cold applications are the best means of controlling a high temperature. They may be in the form of wet packs or an ice-bag over the heart. Just what degree of temperature is an indication for the applications, depends of course, upon the individual case, but as a rule, they should be applied when the temperature reaches 101.

### INDIGESTION.

Minute and particular attention should be paid to every consumptive's diet and to the condition of his digestive organs. When this is done, there will be less serious attacks of indigestion. But in spite of all care these troubles occasionally occur, and often patients come to our hands with digestive organs severely impaired. Careful investigation should be made of when, what and how much the patient is eating and regulations made accordingly. Intelligent individualization of treatment is of importance in this as in all other things. Some patients can digest what others cannot. Some can take more and others need longer intervals. The stomach tube should be used to determine these points, and also to decide whether there is need of giving hydrochloric acid and pepsine, one or both.

Stomach washing gives great help in these stomachic troubles. This may be done once or twice a day, according to the need of the case. If but once, it is best done before breakfast, the stomach being thoroughly cleansed and local applications made if necessary. If it is done before dinner or supper, the preceding meal should have been taken at least five hours before the washing.

Intestinal indigestion is best met by regulating the diet and by washing the intestinal tract as thoroughly as possible each day. Tubercular diarrhœas will usually require drug treatment.

### THE USE OF MEDICINES.

I would not be understood as discarding all drugs. I am not a therapeutic nihilist. If a remedy is clearly indicated, if something is to be gained by its use, then it should be used. But I do protest against multiplicity of doses, and against anything which interferes with digestion and nutrition. And I insist that no drug, or climate, or specific treatment can ever make amends for neglected hygienic management.

